# Supplementary material for: Engineering the spin couplings in atomically crafted spin chains on an elemental superconductor
Source: Nat Commun. 2018 Aug 14;9:3253. doi: 10.1038/s41467-018-05701-8 (PMC6092363; doi:10.1038/s41467-018-05701-8)
Supplement: Supplementary file 1 — Supplementary Information [file 41467_2018_5701_MOESM1_ESM.pdf]

Engineering the spin couplings in atomically crafted spin chains on an elemental superconductor

Kamlapure et al,

Supplementary Figures

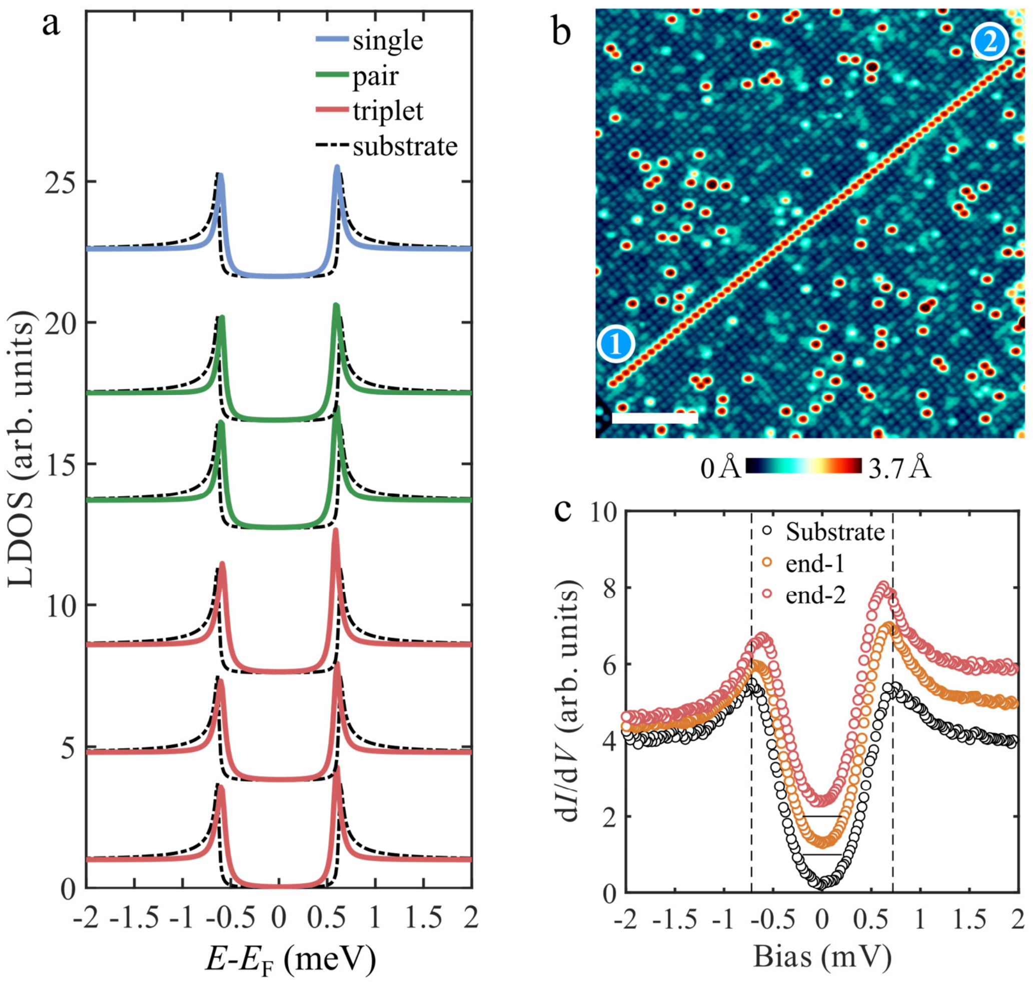

Supplementary Figure 1 | Spectroscopy on different chains. a, The numerically deconvoluted local density of states (LDOS) corresponding to the data in Fig. 1e of the main manuscript. The curves are shifted vertically and grouped for clarity. All the curves are plotted together with the substrate density of states as a reference. Resemblance of these curves indicates the negligible coupling between adatoms in the chains. **b,** Constant-current STM image of the long chain of 63 atoms (same as Fig. 1f in the main manuscript, $V = 100$ mV, $I = 100$ pA). Scale bar is 10 nm. **c,** dI/dV spectra measured with a Cr-tip at the two ends of the chain (end-1 and end-2 shown in **b**) plotted along with the substrate spectrum. For clarity, the spectra are shifted vertically (V_{stab} = 6 mV, I_{stab} = 300 pA, V_{mod} = 100 μV). Spectra taken at the two ends show peaks due to YSR states close to the gap edge. This energetic position of the YSR state is identical to the ones in **a**, and Fig. 2c,g of the main manuscript, showing a negligible coupling along the chain without any prominent features in the spectra.

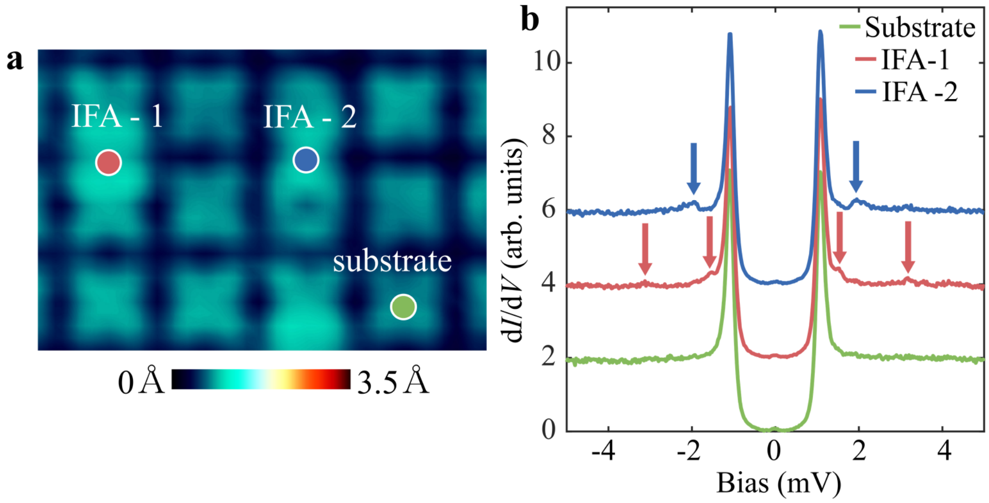

Supplementary Figure 2 | Spectroscopy on the interstitial Fe atoms (IFA). a, Constant-current STM image showing two IFAs which are manipulated in two different surroundings of the superstructures. **b,** dI/dV spectra taken with a superconducting tip on the three locations shown in **a** (V_{stab} = 2.5 mV, I_{stab} = 300 pA, V_{mod} = 20 µV). Spectra are shifted vertically for clarity. Both IFA-1 and IFA-2 show replicas of the coherence peaks (see arrows), possibly arising from a spin excitation[1]. However, the detailed features are different for the two cases, which implies slightly different positions of the IFAs in the interstitial sites. Moreover, the replicas might indicate the presence of a finite magnetic moment of the IFA.

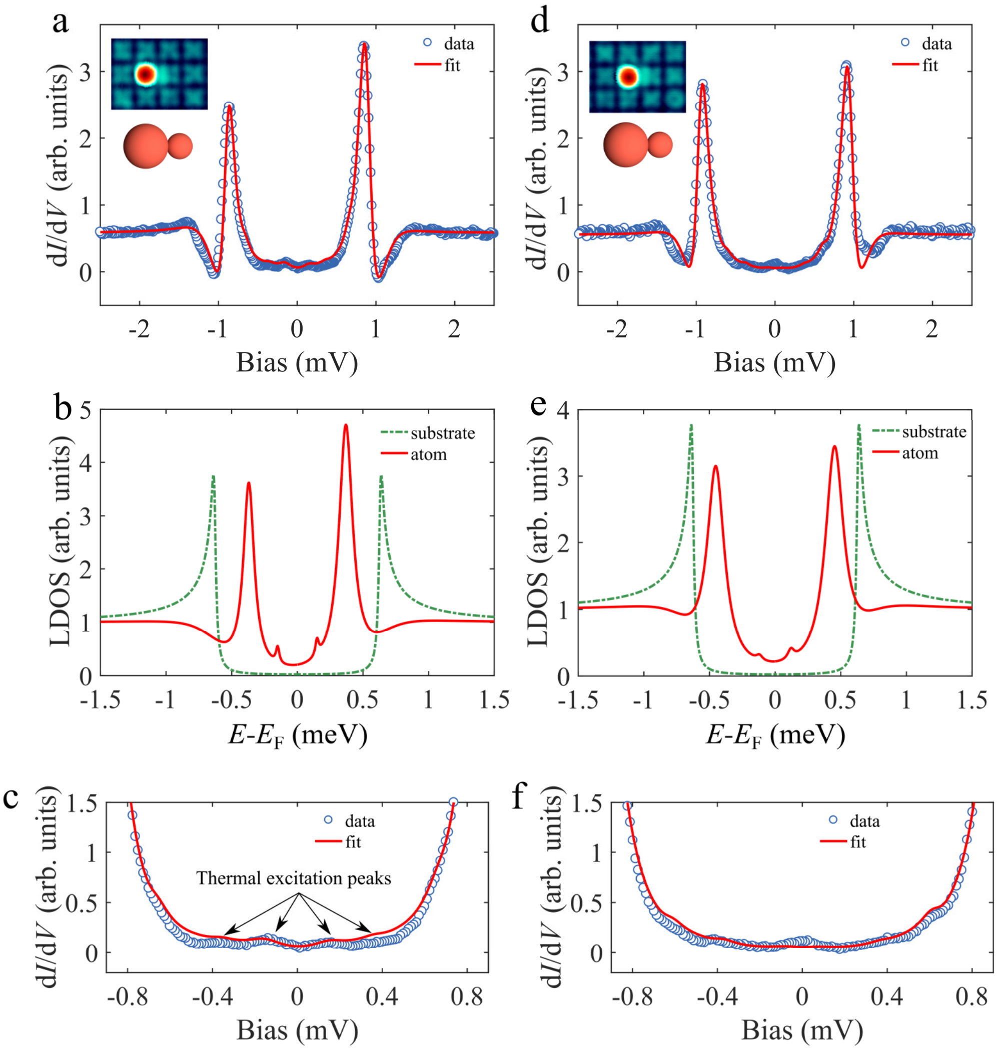

Supplementary Figure 3 | Spectroscopy on an adatom with one IFA in the vicinity. a, dI/dV spectrum taken with a superconducting tip on adatom B (Fig. 3**f** of the main manuscript) plotted together with the fit using numerical deconvolution. The inset shows the STM image of the adatom. The pictogram indicates the positions of the adatom (large sphere) and the IFA (small sphere). **b,** The numerically deconvoluted local density of states (LDOS) on the adatom plotted together with the substrate density of states as a reference. To describe the extra features in the dI/dV spectrum on adatom B (**a**), we need to use multiple YSR states to capture all the spectral features. For a symmetric magnetic dimer with ferromagnetic coupling, four symmetric peaks are expected[2]. In our case, we indeed see two additional peaks, but with a much smaller intensity than that of the main two peaks. **c**, The zoomed-in view of the same spectrum and the fit as in **a**. The fit captures details of the spectral features due to thermally occupied states within the superconducting energy gap of the tip, which are otherwise not prominently visible in **a**. **d-f**, Similar spectral analysis as in **a-c** on another adatom of type B. Again, we need to use multiple YSR states to capture the details of the additional spectral features inside the gap.

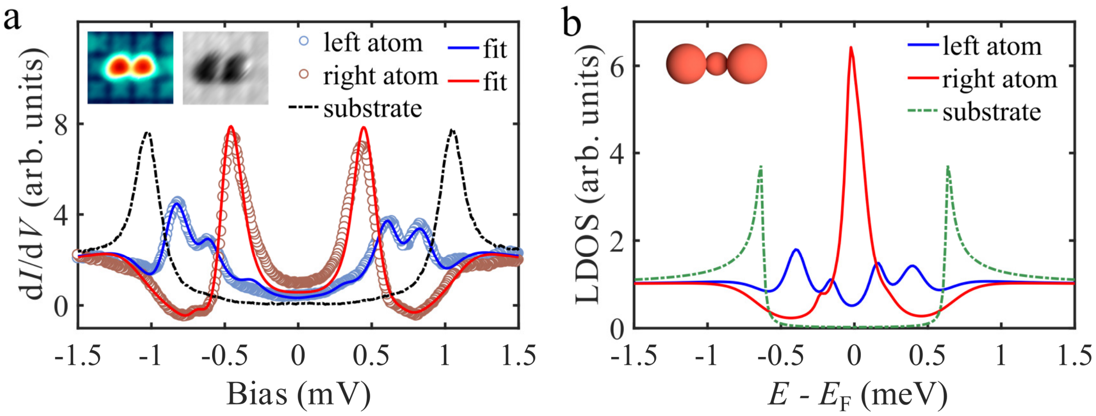

Supplementary Figure 4 | Spectroscopy on a pair with one IFA in the center. a, d*I*/d*V* spectra taken with a superconducting tip on a pair of adatoms with one IFA in the middle, plotted together with the substrate spectrum. Here both of the adatoms are in the YSR-on state. The solid curves represent a fit using the numerical deconvolution. Insets in the panel are the STM image and the d*I*/d*V* image. **b,** The numerically deconvoluted LDOS corresponding to the fits in (a). The substrate spectrum is shown as a reference. The pictogram indicates the positions of the adatoms (large spheres) and the IFA (small sphere). To describe the extra features in the d*I*/d*V* spectrum on each of the atoms in the pair (**a**), we need to use multiple YSR states that capture all the spectral features. This, again, indicates a ferromagnetic coupling[2] to the IFA or between the two adatoms, similar to the structure discussed in Supplementary Figure 3.

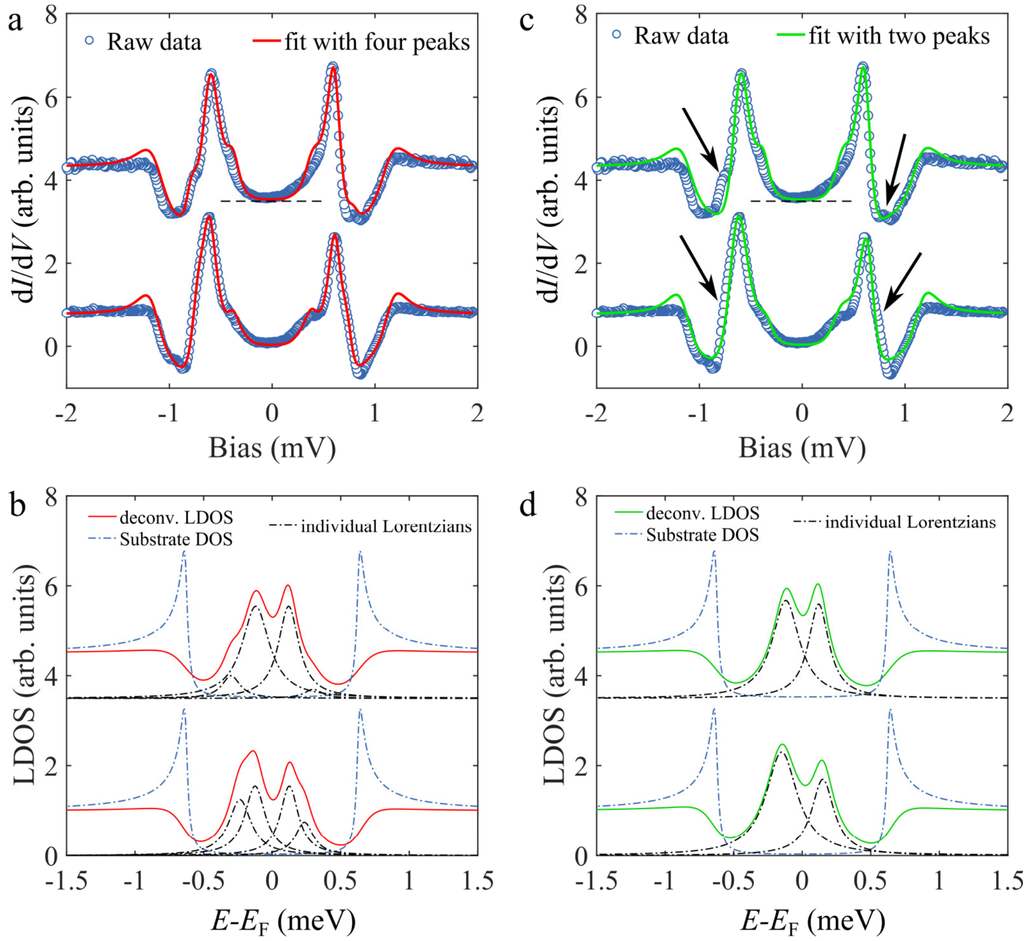

Supplementary Figure 5 | Assessment of the fitting. a, dI/dV spectra (same as Fig. 5e of the main manuscript) taken with a superconducting tip on the 5th atom from the top of the chain, together with the fits from numerical deconvolution of the spectra employing four peaks due to YSR states, in two different situations: (i) all the adatoms in the chain are in YSR-on state (top curves), (ii) 6th atom is in YSR-off state while all others are in YSR-on state (bottom curves). **b,** The numerically deconvoluted LDOS (same as Fig. 5f of the main manuscript) corresponding to the fits in panel **a**. The substrate DOS is plotted as a reference. **c,d**, similar plots as **a**, **b**, respectively, except that in this case only two peaks were employed for fitting the spectra. It is clear from the fits in **c** that the spectral shapes, especially at the positions indicated by black arrows, can be correctly captured only by employing four Lorentzian peaks (see black dash-dotted lines in **b**). This indicates a splitting of the YSR states and implies a spin coupling between the adatoms in the chain.

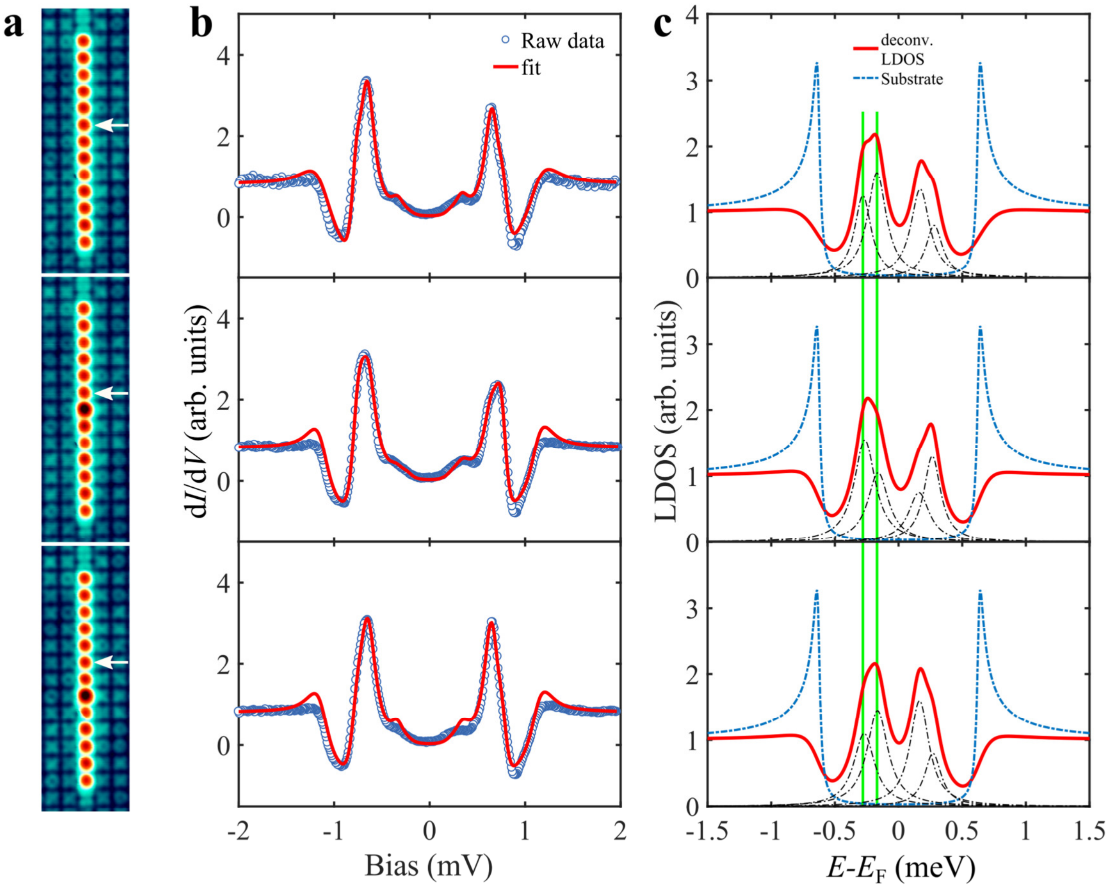

Supplementary Figure 6 | Numerical deconvolution of the spectra on the atom in the chain. a, Constant-current STM topographs of the chain (same chain as shown in Fig.5 of the main manuscript) for the three different situations: all the atoms are in YSR-on state (top), (ii) 7th atom from the top is in YSR-off state while rest of the atoms are in YSR-on state (middle panel), (iii) 8th atom from the top is in YSR-off state while rest of the atoms are in YSR-on state (bottom panel). **b,** dI/dV spectra taken with a superconducting tip on the 6th atom from the top, together with the fits from the numerical deconvolution of the spectrum for the three cases shown in **a**. **c,** The numerically deconvoluted LDOS corresponding to the fits in each panel in **b**. The substrate density of states is plotted as a reference. Vertical green lines in **c** are guides to the eye showing the peak positions of the YSR states in the upper most panel. The deconvoluted LDOS clearly shows that at least four prominent peaks are needed in order to correctly capture the details of the spectra in **b** (see black dash-dotted lines). This indicates that there is a considerable spin coupling between the adatoms in the chain. From the vertical green lines in **c** it is evident, that we do not see significant changes in the separation between the split peaks when switching one of the nearest or next-nearest neighbors into the non-magnetic state. However, the intensity difference between the two peaks on the negative (or positive) bias side is inverted when the 7th atom in the chain is switched to the YSR-off state, and the intensities are regained when the 8th atom is switched to the YSR-off state. This observation is different than the case described in Figure 5**e,f** of the main manuscript, where upon switching the 6th atom from YSR-on to YSR-off state, the separation between the two split YSR states on the neighbouring 5th atom reduces. The different behavior of the split YSR states for the two atoms further confirms an overall inhomogeneous and non-trivial spin-coupling along the chain.

Supplementary References:

1. Heinrich, B. W., Braun, L., Pascual, J. I. & Franke, K. J. Protection of excited spin states by a superconducting energy gap. *Nature Physics* **9,** 765–768 (2013).
2. Flatté, M. E. & Reynolds, D. E. Local spectrum of a superconductor as a probe of interactions between magnetic impurities. *Physical Review B* **61,** 14810–14814 (2000).
